# Supplementary material for: Potential of Human Hemoglobin as a Source of Bioactive Peptides: Comparative Study of Enzymatic Hydrolysis with Bovine Hemoglobin and the Production of Active Peptide α137–141
Source: Int J Mol Sci. 2023 Jul 25;24(15):11921. doi: 10.3390/ijms241511921 (PMC10418852; doi:10.3390/ijms241511921)
Supplement: Supplementary file 1 [file ijms-24-11921-s001.zip › ijms-2491502-supplementary.pdf]

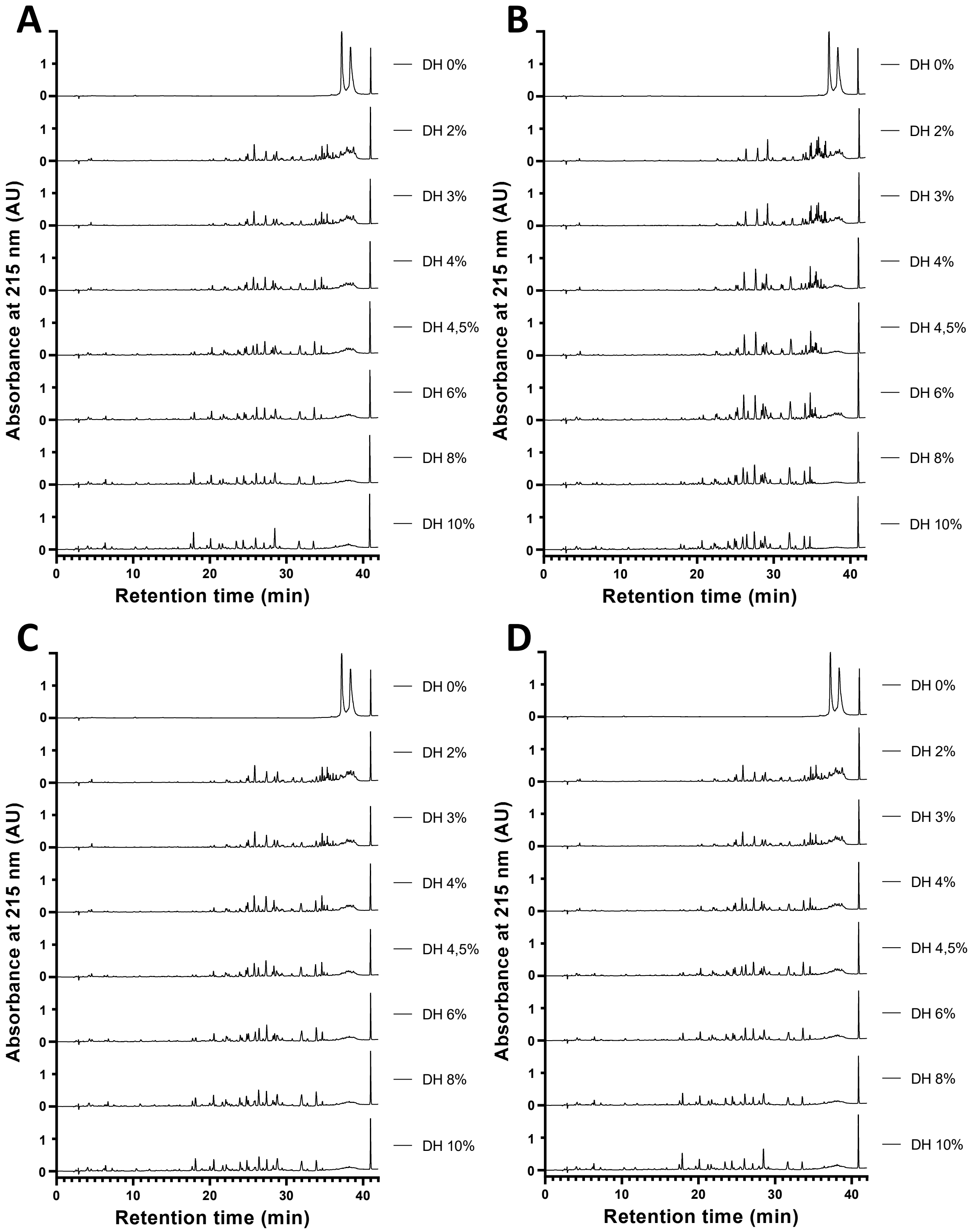

(1) *Bovine hemoglobin*

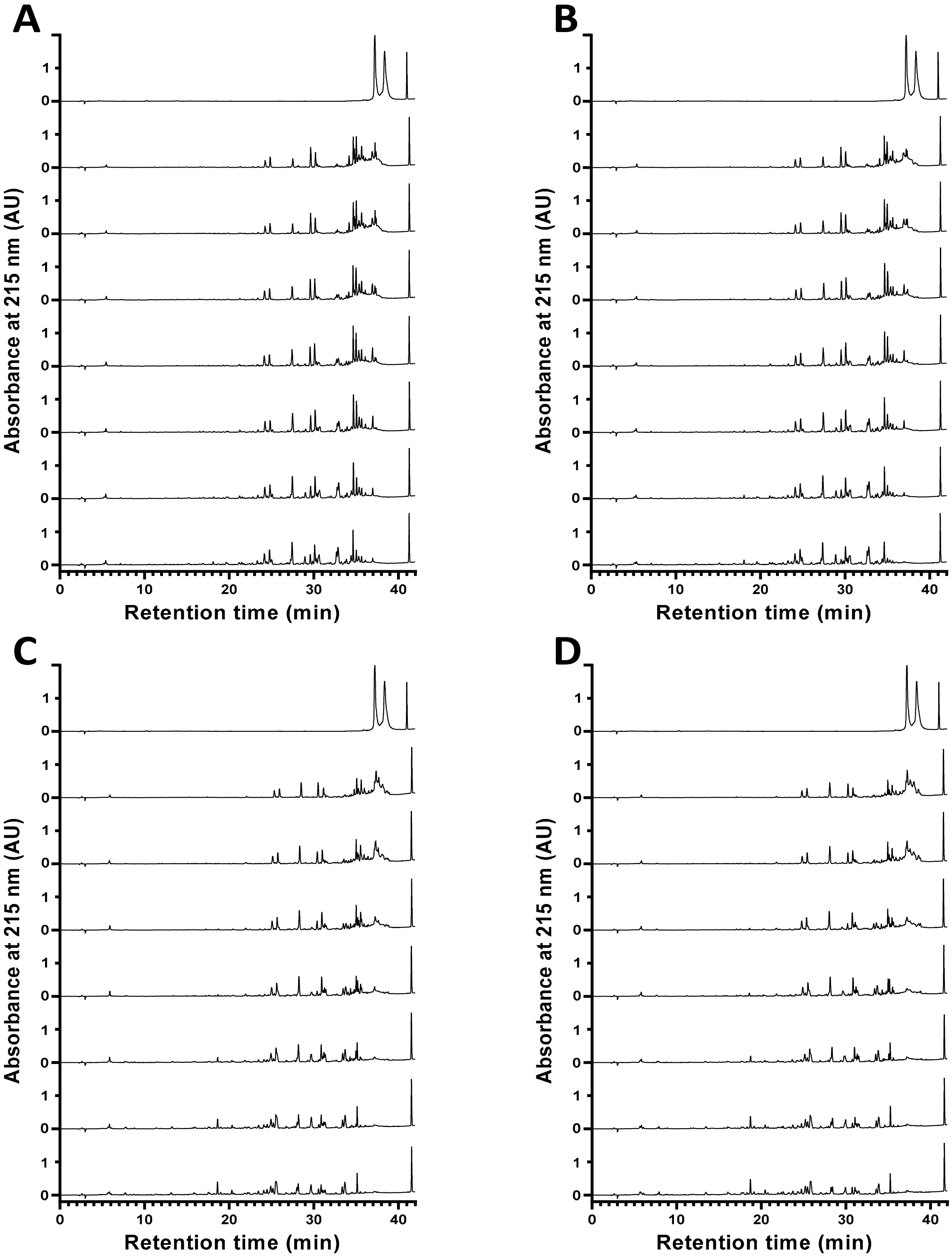

(2) *Human hemoglobin*

Figure S1: Chromatographic hydrolysis profiles of bovine hemoglobin (1) and human hemoglobin (2) at 215 nm by RP-UPLC, analyzed by C4 type column for different initial concentrations of bovine and human hemoglobin (pH 3.5, 23°C, E/S = 1/11) (A) 1%, (B) 2% diluted by 2, (C) 8% diluted by 8, (D) 10% diluted by 10
